# Predicting outcome after aneurysmal subarachnoid hemorrhage by exploitation of signal complexity: a prospective two-center cohort study

**DOI:** 10.1186/s13054-024-04939-7

**Published:** 2024-05-14

**Authors:** Stefan Yu Bögli, Ihsane Olakorede, Michael Veldeman, Erta Beqiri, Miriam Weiss, Gerrit Alexander Schubert, Jan Folkard Willms, Emanuela Keller, Peter Smielewski

**Affiliations:** 1 Brain Physics Laboratory, Division of Neurosurgery, Department of Clinical Neurosciences, University of Cambridge, Cambridge, UK; 2 Neurocritical Care Unit, Institute for Intensive Care and Department for Neurosurgery, University Hospital Zurich, University of Zurich, Zurich, Switzerland; 3 Department of Neurosurgery, RWTH Aachen University, Aachen, Germany; 4 Department of Neurosurgery, Cantonal Hospital Aarau, Aarau, Switzerland

**Keywords:** Complexity, Multiscale entropy, Subarachnoid hemorrhage, Outcome

## Abstract

**Background:**

Signal complexity (i.e. entropy) describes the level of order within a system. Low physiological signal complexity predicts unfavorable outcome in a variety of diseases and is assumed to reflect increased rigidity of the cardio/cerebrovascular system leading to (or reflecting) autoregulation failure. Aneurysmal subarachnoid hemorrhage (aSAH) is followed by a cascade of complex systemic and cerebral sequelae. In aSAH, the value of entropy has not been established yet.

**Methods:**

aSAH patients from 2 prospective cohorts (Zurich—derivation cohort, Aachen—validation cohort) were included. Multiscale Entropy (MSE) was estimated for arterial blood pressure, intracranial pressure, heart rate, and their derivatives, and compared to dichotomized (1–4 vs. 5–8) or ordinal outcome (GOSE—extended Glasgow Outcome Scale) at 12 months using uni- and multivariable (adjusted for age, World Federation of Neurological Surgeons grade, modified Fisher (mFisher) grade, delayed cerebral infarction), and ordinal methods (proportional odds logistic regression/sliding dichotomy). The multivariable logistic regression models were validated internally using bootstrapping and externally by assessing the calibration and discrimination.

**Results:**

A total of 330 (derivation: 241, validation: 89) aSAH patients were analyzed. Decreasing MSE was associated with a higher likelihood of unfavorable outcome independent of covariates and analysis method. The multivariable adjusted logistic regression models were well calibrated and only showed a slight decrease in discrimination when assessed in the validation cohort. The ordinal analysis revealed its effect to be linear. MSE remained valid when adjusting the outcome definition against the initial severity.

**Conclusions:**

MSE metrics and thereby complexity of physiological signals are independent, internally and externally valid predictors of 12-month outcome. Incorporating high-frequency physiological data as part of clinical outcome prediction may enable precise, individualized outcome prediction. The results of this study warrant further investigation into the cause of the resulting complexity as well as its association to important and potentially preventable complications including vasospasm and delayed cerebral ischemia.

**Supplementary Information:**

The online version contains supplementary material available at 10.1186/s13054-024-04939-7.

## Introduction

Aneurysmal subarachnoid hemorrhage (aSAH) remains a serious disease with often poor prognosis even after successful securing of the aneurysm [1]. Patients who survive the initial hemorrhage remain at risk for developing secondary brain injury, such as delayed cerebral ischemia (DCI) [2]. DCI is a major cause of death and disability after aSAH [3]. It is the consequence of complex interactions of neuronal activity, cerebral and systemic hemodynamics, and feedback mechanisms—neurovascular (un)coupling, cerebral autoregulation, and CO_2_ reactivity [2]. Dynamic changes of multiple interacting factors including cerebral vasospasm [4], inflammatory markers [5], oxygenation [6], blood pressure, and cardiac output [7] precede DCI occurrence. The paramount goal of neurocritical care is to predict, counteract or even prevent these secondary injuries to improve patients’ outcome. Consequently, the acute period following the hemorrhage is accompanied by extensive multimodal monitoring within a neurocritical care unit (NCCU) environment. The monitoring comprises aspects of cerebral physiology and hemodynamics (incl. intracranial pressure (ICP), cerebral perfusion pressure (CPP)) integrated with systemic physiological parameters (arterial blood pressure, cardiac output, heart rate (HR), oxygenation, and ventilation) [8].

Signal complexity (i.e. entropy) describes the level of apparent disorder within a system. Low signal complexity predicts unfavorable outcome in a variety of diseases and is assumed to reflect increased rigidity of the cardio/cerebrovascular feedback/regulating system leading to (or reflecting) autoregulation failure [9–12] This, in turn, leaves the brain susceptible to secondary injury. Physiological systems are regulated by multiple, interacting, mechanisms leading to dynamically changing biosignals across different temporal scales.^14^ Multiscale entropy (MSE), a version of signal complexity, estimates sample entropy over a range of increasingly downsampled (i.e. averaged) data [13, 14] In comparison to sample entropy of a single scale MSE has the following benefits: 1. It allows for the evaluation of complex physiological systems that operate across different time scales; 2. It suppresses the impact of noise on the resulting metric. In 2012 Lu et al. described the association between decreased ICP signal complexity and unfavorable outcome after traumatic brain injury [9]. Zeiler et al. validated the concepts presented in a large multi-center cohort and extended the description to include other biosignals [15]. In aSAH, a metric related to signal complexity, heart rate variability, has shown, to a degree, an association with complications and unfavorable outcome [16–18]. However, other patho-physiological states such as sepsis decrease heart rate variability, whereby its use in clinical practice for prediction of specific complications has remained limited. We aimed to exploit the abundance of monitoring data acquired from each patient within an NCCU environment to assess the potential use of MSE as an outcome predictor after aSAH.

## Materials and methods

The study was approved by the local ethics committees Zurich and Aachen and was in accordance with the ethical standards laid down in the 2013 Declaration of Helsinki for research involving human subjects. Informed consent was received before inclusion by the patient or their legal medical representative. Data from two prospective observational cohorts (University Hospital Zurich, Switzerland; the Rheinisch-Westfälische Technische Hochschule Aachen, Germany) was analyzed. The Zurich cohort was used as the derivation cohort to establish models and analyses, while the Aachen cohort was used for external validation.

### Study population

For the Zurich cohort a total of 244 consecutively admitted adult patients with aSAH were recruited as part of the *ICU Cockpit Prospective Cohort Study* between 2016 and 2022. All of these received multimodal monitoring data acquisition and were consequently evaluated for inclusion. For the Aachen cohort a total of 316 consecutively admitted adult patients with aSAH were collected as part of a prospective cohort between 2014 and 2021. 102 of these received multimodal monitoring data acquisition and were consequently evaluated for inclusion. Inclusion criteria were: 1. aSAH due to an angiography confirmed ruptured aneurysm; 2. Admission to the NCCU and recording of high-resolution monitoring data. The only exclusion criterion was loss to follow up with missing 12-month outcome. Patients at both centers were treated according to the guidelines of the Neurocritical Care Society, American Heart Association guidelines, and the respective standard therapies of the two centers [19, 20].

### Data acquisition

The following relevant clinical data were prospectively included in the respective databases: Demographics, World Federation of Neurological Surgeons scale (WFNS)[21], modified Fisher Score (mFisher) [22], clinical course incl. aneurysm occlusion modality, occurrence of angiographic vasospasm (defined as narrowing of the vessels in neuroimaging independent of clinical symptoms), delayed cerebral infarction (DCI—infarction within neuroimaging not present on imaging performed within 24–48 h after aneurysm occlusion, and not attributable to other causes [23]), and outcome at 12 months (represented by the Glasgow Outcome Scale Extended—GOSE [24]). WFNS was evaluated after neurological resuscitation (i.e. after insertion of EVD and/or hematoma evacuation). In either center outcome was assessed during routine outpatient follow-up consultations or by contacting the patient, their next of kin, or caregiver by telephone in a structured interview. Physiological high-resolution data (at least 100 Hz—BP, ICP, HR) was collected in Zurich (Moberg Component Neuromonitoring Systems (CNS)—Moberg Research Inc, PA, USA) and Aachen (MPR2 logO Datalogger (Raumedic, Helmbrechts, Germany) or, after July 2018, Moberg Component Neuromonitoring Systems (CNS)—Moberg Research Inc, PA, USA). The data acquisition was started after admission to the respective NCCUs (after neurological resuscitation and generally after securing of the aneurysm) and stopped either when the patient was discharged to the ward or if invasive monitoring was deemed unnecessary.

### Data preprocessing

The high-resolution (i.e. waveform) monitoring data from either center was transformed into an HDF5 format for streamlined analysis of the different formats. NCCU high-resolution waveform data contains, without exception, artifacts which are not representative of the patients’ physiology. Thus, raw waveform data was preprocessed using ICM + ® (Cambridge Enterprise Ltd, Cambridge, United Kingdom). Data was curated to remove artifacts using both manual and automated methods. The manual methods were applied to remove sections with arterial line failure (continuous reduction of the arterial blood pressure amplitude followed by flushing) and sections with manipulation or opening of the external ventricular drain (EVD—high frequency artefacts with or without sudden changes of ICP level). Automated methods for cleaning of arterial blood pressure were removal of pressure below 0 or above 300 mmHg and removal of sections with pulse amplitude of less than 15 mmHg. Automated methods for cleaning of ICP included removal of values below − 20 or above 200 mmHg, removal of sections with low amplitude (< 0.04 mmHg) corresponding to noise or EVD opening, and removal of values with a 95% Spectral edge frequency above 10 Hz (high-frequency noise). Only the remaining data (termed artifact-free) is used for further processing mitigating the effect of artificial, non-physiological sections.

Data was then processed to acquire 10 s averages of mean arterial blood pressure (ABP), systolic blood pressure (SBP), diastolic blood pressure (DBP), ICP, ICP amplitude (AMP), CPP (difference between ABP and ICP), and HR. Averaging, in effect, allowed for the removal of cardiac and respiratory components.

### Multiscale entropy analysis

MSE was calculated as previously described based on the estimation of sample entropy [13]. Sample entropy describes the probability that matching sequences of length *m* will exhibit the same behavior (i.e. will also match) when extended by one point. It is estimated as the negative natural logarithm of the ratio between the number of *m* + 1 length patterns to the corresponding m length patterns [25]. We estimated sample entropy using m = 2 and a tolerance of 0.15. MSE describes the process of calculating sample entropy over different time scales. A total of 20 scales starting from 1 up to 20 (produced by averaging based coarse graining i.e. Step 1—no averaging, step 2—averaging of 2 consecutive samples … step 20—averaging of 20 consecutive samples) covering the range of slow waves was used. MSE is the resulting area under the curve (AUC) of the plotted sample entropies. Higher values represent higher signal entropy/complexity. MSE was calculated for each of the 10 s biosignals resulting in the metrics MSE abp, MSE sbp, MSE dbp, MSE cpp, MSE hr, MSE icp, MSE amp.

### Statistical analysis

Statistical analysis was performed in R Studio (R version 4.3.2—https://www.r-project.org/—packages used: *rstatix, pROC, boot, rms, MASS, ResourceSelection, predtools, brant*).

Descriptive variables are reported as counts/percentages or mean ± standard deviation. Distribution of the different continuous variables was assessed using the Shapiro–Wilk test. Equality of variances was tested using the Bartlett test or the Levene test. Different statistical methods were explored to assess the association between MSE and outcome. Both univariable as well as multivariable analysis (covariates: age, WFNS, mFisher, and occurrence of DCI) were performed. A significance level of *p* < 0.05 was set due to the exploratory nature of the study and the different tests used for exploration. The Bonferroni corrected adjusted significance level would be *p* = 0.00089.

Univariable: First the different MSE variables were compared to outcome as dichotomized by GOSE (1–4 vs. 5–8) using independent t-tests. To assess the overall diagnostic performance of the different MSE metrics, ROC curves (receiver operating characteristic curves) were plotted and evaluated by calculating the AUC (overall diagnostic performance) and its confidence interval (CI), and by estimating the optimal threshold (based on the Youden Index) to assess sensitivity, specificity, positive/negative predictive values, and accuracy. MSE metrics were then plotted against outcome as grouped into Dead/Vegetative (GOSE 1–2), Severe Disability (GOSE 3–4), Moderate Disability (GOSE 5–6), and Good Recovery (GOSE 7–8) and evaluated by analysis of variance (ANOVA).

Multivariable: Covariate adjusted logistic regression models were built with dichotomized GOSE (1–4 vs. 5–8) as endpoint to assess the independence of the MSE metrics as predictors of outcome. Effect of the metrics on model performance was described using the odds ratio (OR) including its CI. Diagnostic performance of the models was assessed using the AUC, the Nagelkerke R^2^ (R^2^), and the Brier Score. The effect of MSE metric inclusion was evaluated using the DeLong’s test comparing the different AUCs to a base model without the inclusion of MSE metrics. The established models were validated both internally as well as externally. Internal validation was performed by bootstrapping (1000 replications with replacement). During this process prediction models were derived from each bootstrap sample and applied to both the bootstrap and the original dataset allowing for the estimation of optimism (i.e. the difference between the AUC/R^2^/Brier scores of the results derived from the original vs. the different bootstrapping data sets). External validity was assessed by: 1. Evaluating the calibration (agreement between predicted and observed outcome described using its intercept and slope and assessed using the Hosmer–Lemeshow-goodness-of-fit test) 2. Evaluating the discrimination (AUC) when applying the derivation-dataset-based model to the validation cohort.

Ordinal multivariable: Due to the ordinal nature of the outcome score we additionally performed a proportional odds logistic regression and a sliding dichotomy analysis. Both, proportional odds logistic regression as well as sliding dichotomy allow for exploiting the range of the outcome scale by providing either the assessment of OR across different cutoffs or the assessment of baseline adjusted outcome definitions thereby increasing statistical power [26]. Proportional odds logistic regression adjusted for covariates was applied to the same scales as described above with moving cutoffs (Dead/Vegetative vs. Severe Disability, Severe Disability vs. Moderate Disability, Moderate Disability vs. Good Recovery) to assess the common odds ratio. The proportional odds assumption was tested using the Brant-Wald test. Lastly a sliding dichotomy approach was used to assess the importance of MSE metrics for a baseline severity adjusted outcome definition. For each patient, based on the baseline covariates (age, WFNS, mFisher score, and occurrence of DCI), a prognostic risk probability for unfavorable outcome was estimated. The resulting scores were then divided into 3 prognostic groups of roughly equal size corresponding to low, intermediate, and high likelihood of unfavorable outcome. For each prognostic group a separate cutoff was defined to dichotomize outcome into favorable and unfavorable, with the adjusted favorable outcome classified as:

- GOSE 7–8: for the group with low likelihood for unfavorable outcome,
- GOSE 5–8: for the group with intermediate likelihood for unfavorable outcome
- GOSE 3–8: for the group with high likelihood for unfavorable outcome.

The resulting baseline severity adjusted outcome variable was then assessed against the MSE metrics using logistic regression. For both methods bootstrapping was applied for internal validation and to acquire the CI.

### Secondary analysis

Three additional secondary analyses were performed to assess further aspects associated with the metric MSE based on the most promising metrics found. First: To assess, whether early outcome prediction using MSE is feasible, a secondary analysis was performed including only data acquired within the first 48 h after NCCU admission. Second: To evaluate whether MSE was associated with specific clinical aspects of the disease, values were assessed against clinical events. For this purpose, the following additional clinical parameters were extracted from the electronic patient records (occurrence of rebleeding, global cerebral edema, brain herniation, and seizures) and evaluated using t-tests. The raw metrics (ABP, HR, ICP) were assessed against the derived MSE metric to reveal possible intercorrelations. Third: The stability of the metric was assessed by evaluating the change when considering longer amounts of data within one patient (between 1 and 24 h) as well as when comparing the results of the metrics to the duration of the measurement in the whole cohort.

## Results

### Patient characteristics and high-resolution data availability

**Fig. 1:**
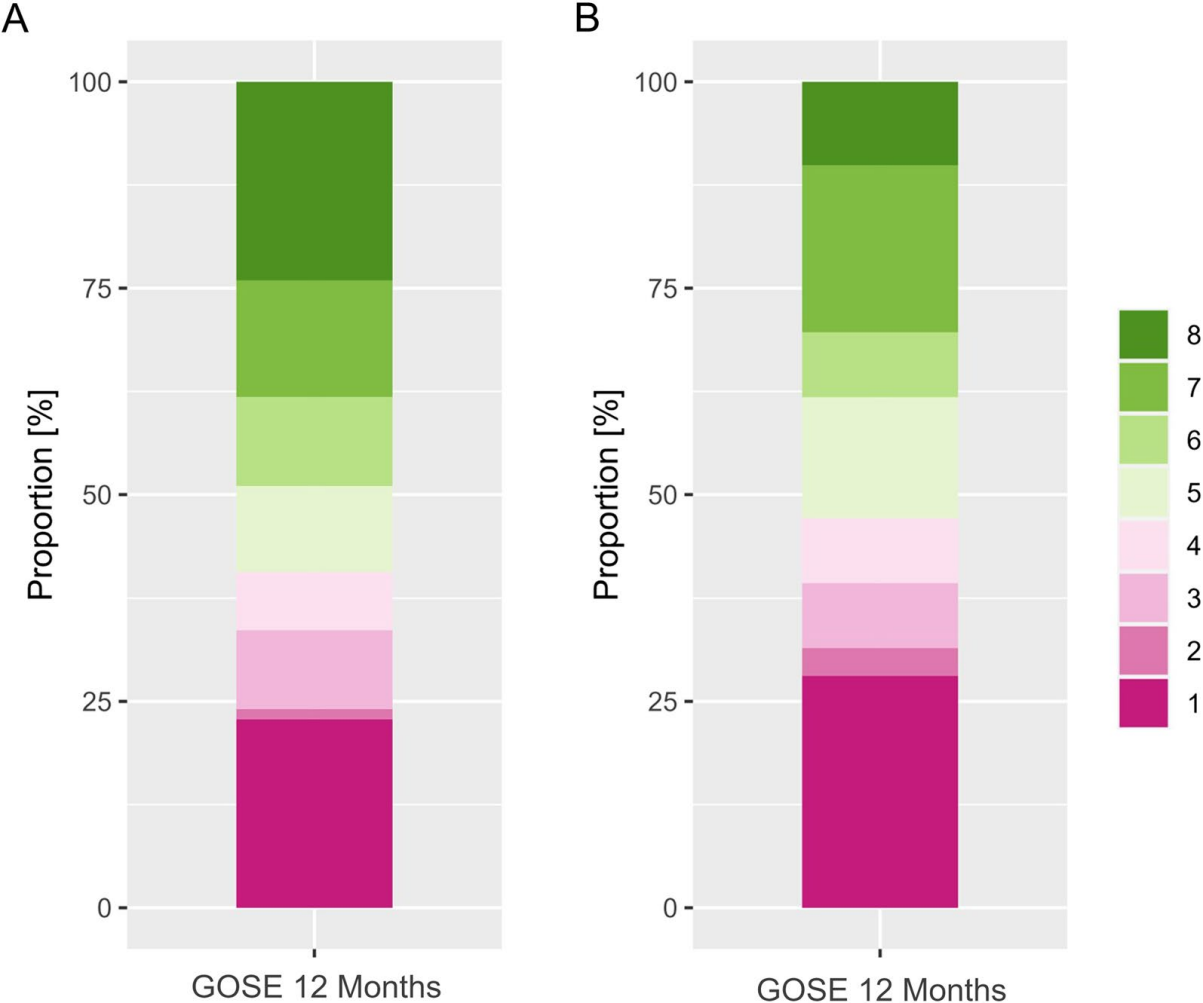
Glasgow Outcome Scale Extended (GOSE) at 12 months (**A**—derivation cohort; **B**—validation cohort)

Derivation cohort: 241 patients were included as part of the derivation cohort (3 were excluded due to loss to follow up). ABP/HR data was available in all patients, ICP data was available in 150 (62%) patients. The following amount of artefact free data was available: ABP—239 h/patient (total of 57′257 monitoring hours), HR—267 h/patient (total of 63′955 monitoring hours), ICP—205 h/patient (total of 30′778 monitoring hours). Validation cohort: 89 Patients were included as part of the derivation cohort (13 were excluded due to loss to follow up). ABP/HR data was available in 101 (99%), and ICP data was available in 73 (72%) patients. The following amount of artefact free data was available: ABP/HR—268 h/patient (total of 23′553 monitoring hours), ICP—290 h/patient (total of 21′169 monitoring hours). The median time between the initial hemorrhage and start of multimodality monitoring was 18 h in the derivation and 31 h in the validation cohort. The distributions of available data of the derivation and validation cohort can be found in the supplement including overall lengths of datasets as well as the density with respect to the timing from the initial hemorrhage (Additional file 1: A). The highest density of data was available between day 3 and 14 after the initial hemorrhage in either center. Overall descriptions of physiology metrics can be found in the supplement (Additional file 1: B). As this was a cohort undergoing active treatment, ICP within either cohort was mostly below 20 mmHg and ABP was around 90–100 mmHg. The clinical characteristics of the derivation and validation cohort can be found in Table 1. The outcome at 12 months (assessed using GOSE) is shown in Fig. 1.

**Table 1:** Patient characteristics^a^

	Derivation cohort (Zurich)	Validation cohort (Aachen)
Patients (n)	N = 241	N = 89
Age (years)	58 ± 12.9	56 ± 10.7
Sex (female)	175 (65%)	63 (71%)
Aneurysm location (Anterior circulation)	183 (76%)	77 (87%)
*WFNS*		
1	74 (31%)	20 (23%)
2	46 (19%)	14 (16%)
3	17 (7%)	21 (24%)
4	52 (22%)	22 (25%)
5	52 (22%)	12 (14%)
*mFisher*		
1–2	14 (6%)	32 (36%)
3	69 (29%)	28 (32%)
4	158 (66%)	29 (32%)
*Treatment*		
Clipping	106 (44%)	37 (42%)
Coiling	115 (48%)	52 (58%)
Other	20 (8%)	0 (0%)
Hydrocephalus	141 (42%)	56 (63%)
Vasospasm	156 (65%)	39 (44%)
DCI	62 (26%)	24 (27%)

^a^*WFNS* World Federation of Neurosurgical Societies grading scale, *mFisher* modified Fisher scale, *DCI* delayed cerebral infarction

### Univariable analysis

**Fig. 2:**
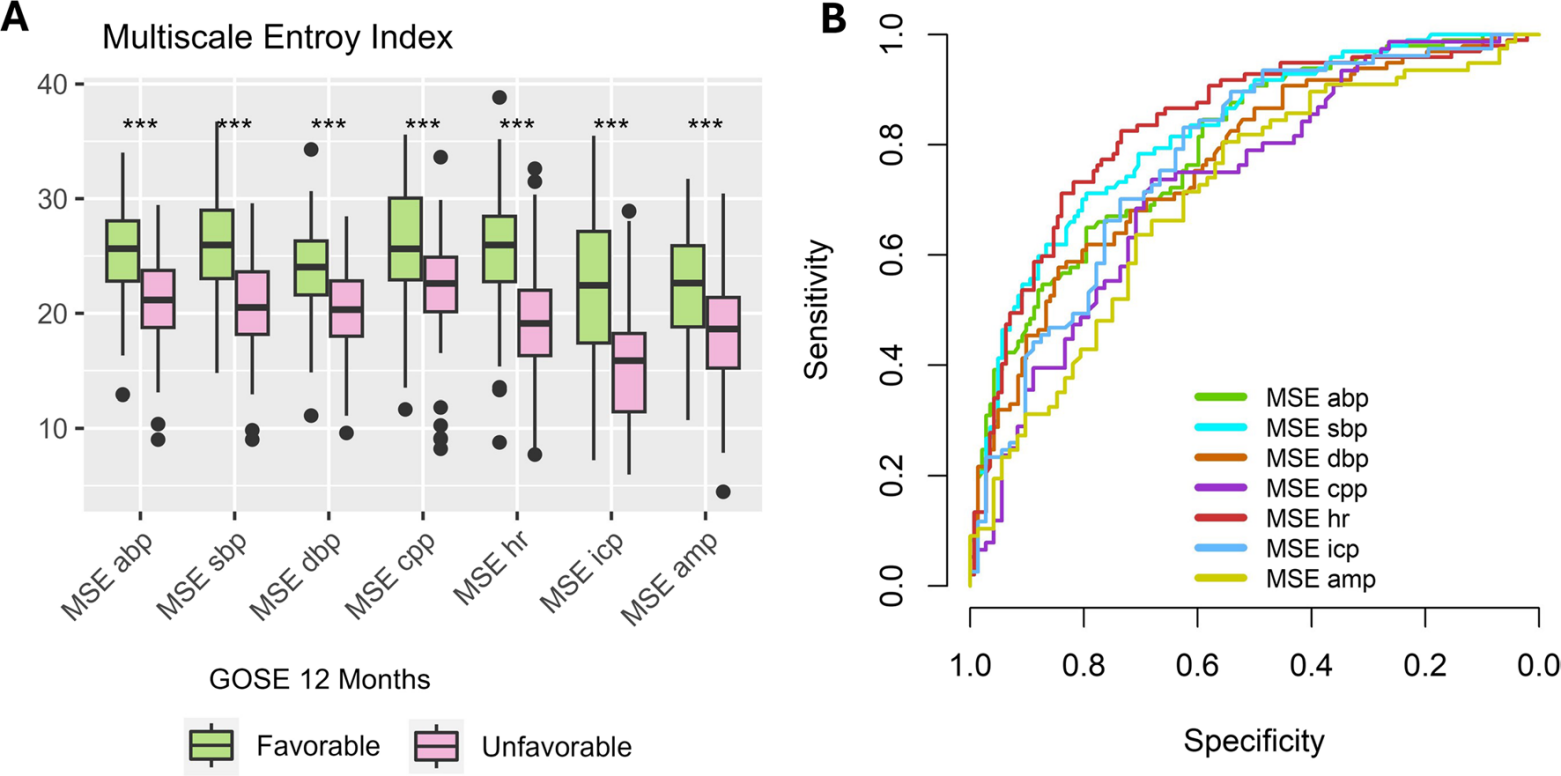
MSE vs. Dichotomized Outcome. Panel A. The different MSE metrics are shown using boxplots comparing unfavourable (GOSE 1–4; pink) and favourable (5–8; green) outcome. An independent t-test was used for statistical analysis. Significant differences are shown using asterisks (*** = *p* < 0.001). The specific *p*-values were: MSE abo (9.15 e-16), MSE sbp (3.81 e-19), MSE dbp (7.17 e-13), MSE cpp (1.77 e-6), MSE hr (6.65 e-19) MSE icp (4.70 e-10), MSE amp (6.72 e-6). Panel B shows the corresponding ROC curves describing the predictive value of the different MSE scores

**Fig. 3:**
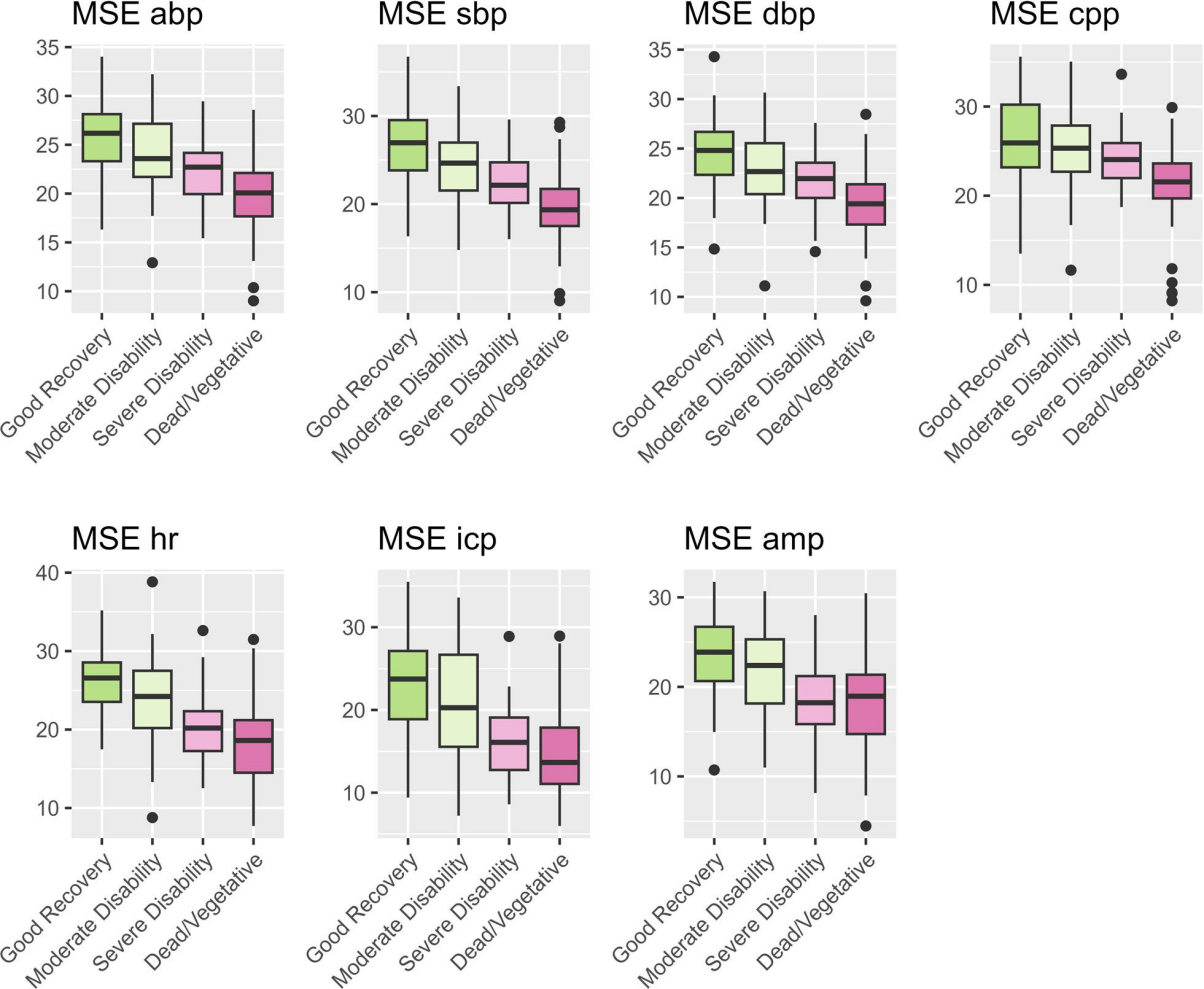
MSE vs. Ordinal Outcome. The different MSE metrics are shown using boxplots grouped by ordinal outcome: Dead/Vegetative (GOSE 1–2), Severe Disability (GOSE 3–4), Moderate Disability (GOSE 5–6), and Good Recovery (GOSE 7–8). The color coding ranges from intense pink (dead/vegetative) to intense green (good recovery). The respective p-values of the performed ANOVA can be found in Table 3

In a first step, the different MSE metrics were evaluated against dichotomized outcome (GOSE 1–4 vs. GOSE 5–8). Overall, there was a difference between the outcome groups irrespective of the MSE metric. The specific *p*-values were: MSE abp (9.15 e-16), MSE sbp (3.81 e-19), MSE dbp (7.17 e-13), MSE cpp (1.77 e-6), MSE hr (6.65 e-19) MSE icp (4.70 e-10), MSE amp (6.72 e-6). The respective data is shown in form of boxplots in Fig. 2 panel A. The predictive value of the different metrics (ROC curves) is shown in Fig. 2 panel B. The specific AUC (CI) can be found in Table 2. AUC ranged between 0.71 and 0.83. The highest values were found for MSE hr (AUC 0.83 (0.78–0.89)) and MSE sbp (AUC 0.82 (0.77–0.87)). The Youden Index was established for each MSE metric and used to calculate related metrics and accuracy (Table 2). The accuracy of the metrics was between 68% (MSE amp) and 77% (MSE hr) when using the Youden Index as a cutoff. To assess the MSE metrics against a higher granularity of outcome, they were then plotted against Dead/Vegetative, Severe Disability, Moderate Disability, and Good Recovery (Fig. 3). The respective *p*-values can be found in Table 3. Overall, there were monotonic decreases of MSE with higher values found in more favorable outcomes.

**Table 2:** The AUCs and Youden Index based thresholds^a^

MSE	AUC (95%-CI)	Threshold	Sensitivity (%)	Specificity (%)	PPV (%)	NPV (%)	Accuracy (%)
abp	0.80 (0.74–0.85)	22.3	65	80	68	77	74
sbp	0.82 (0.77–0.87)	22.7	71	80	70	80	76
dbp	0.77 (0.71–0.83)	20.6	58	85	72	75	74
cpp	0.73 (0.65–0.81)	24.6	74	68	71	71	71
hr	0.83 (0.78–0.89)	23.0	82	73	68	86	77
icp	0.77 (0.70–0.85)	19.6	83	62	70	78	73
amp	0.71 (0.62–0.79)	22.2	81	56	66	73	68

Based on the univariable MSE ROC the different AUCs as well as the corresponding Youden Index based optimal threshold, sensitivity, specificity, positive and negative predictive values, overall accuracy are shown

^a^*ABP* mean arterial blood pressure, *AMP* intracranial pressure amplitude, *AUC* area under the curve, *CI* confidence interval, *CPP* cerebral perfusion pressure, *DBP* diastolic blood pressure, *HR* heart rate, *ICP* intracranial pressure, *MSE* multiscale entropy, *NPV* negative predictive value, *PPV* positive predictive value, *SBP* systolic blood pressure

**Table 3:** *P* values of ANOVA comparing ordinal outcome and MSE metrics^a^

	MSE abp	MSE sbp	MSE dbp	MSE cpp	MSE hr	MSE icp	MSE amp
Good vs. Moderate	0.037	0.0087	0.075	0.526	0.018	0.233	0.544
Good vs. Severe	1.70 e-6	3.74 e-8	1.64 e-4	0.069	7.69 e-10	2.11 e-5	0.0018
Good vs. Dead/Vegetative	4.19 e-14	2.78 e-14	4.84 e-14	4.86 e-7	0.00e+00	5.82 e-9	2.69 e-4
Moderate vs. Severe	0.057	0.028	0.252	0.658	0.002	0.020	0.087
Moderate vs. Dead/Vegetative	4.42 e-8	9.17 e-10	1.20 e-6	3.45 e-4	1.78 e-8	1.01 e-4	0.035
Severe vs. Dead/Vegetative	0.018	0.0059	0.013	0.035	0.192	0.649	0.990

^a^Good—Good Recovery (GOSE 7–8); Moderate—Moderate Disability (GOSE 5–6), Severe—Severe Disability (GOSE 3–4); Dead/Vegetative—Dead/Vegetative (GOSE 1–2)

### Multivariable analysis

To assess the independence of MSE metrics when corrected for covariates, multivariable logistic regression models were built. The results describing adjusted effect of the metric (OR), as well as the overall model performance (AUC, Nagelkerke R^2^, Brier Score) are shown in Table 4 (top panel). All MSE metrics remained independently associated with outcome with OR between 0.78 (MSE sbp) and 0.86 (MSE amp). AUCs ranged between 0.79 and 0.87 and R^2^ between 0.32 and 0.51. Overall MSE sbp and MSE hr showed the highest effect and discriminatory value. MSE abp (*p* = 0.0068), MSE sbp (*p* = 0.0028), MSE dbp (*p* = 0.024), MSE cpp (*p* = 0.032), MSE hr (*p* = 0.003), MSE icp (*p* = 0.004), MSE amp (*p* = 0.029) all increased the AUC when compared to the model excluding the MSE metrics.

**Table 4:** Covariate adjusted logistic regression with internal and external validation.^a^

Original	MSE abp	MSE sbp	MSE dbp	MSE cpp	MSE hr	MSE icp	MSE amp
OR (CI)	0.79 (0.71–0.86)	0.78 (0.70–0.85)	0.81 (0.73–0.89)	0.85 (0.77–0.93)	0.80 (0.74–0.86)	0.84 (0.78–0.90)	0.86 (0.80–0.93)
AUC (CI)	0.85 (0.81–0.90)	0.86 (0.82–0.91)	0.84 (0.79–0.89)	0.79 (0.72–0.86)	0.87 (0.83–0.92)	0.83 (0.76–0.89)	0.79 (0.72–0.87)
R^2^	0.47	0.50	0.44	0.32	0.51	0.40	0.33
Brier Score	0.15	0.15	0.16	0.19	0.14	0.17	0.18

Internal validation	MSE abp	MSE sbp	MSE dbp	MSE cpp	MSE hr	MSE icp	MSE amp
AUC	0.85	0.86	0.84	0.78	0.87	0.82	0.78
R^2^	0.46	0.49	0.42	0.30	0.50	0.38	0.31
Brier Score	0.16	0.15	0.16	0.20	0.15	0.18	0.19

External validation	MSE abp	MSE sbp	MSE dbp	MSE cpp	MSE hr	MSE icp	MSE amp
AUC (CI)	0.76 (0.65–0.86)	0.76 (0.65–0.86)	0.76 (0.66–0.87)	0.72 (0.60–0.84)	0.79 (0.69–0.89)	0.80 (0.70–0.90)	0.76 (0.65–0.87)
Hosmer–Lemeshow-goodness-of-fit	*p* = 0.18	*p* = 0.07	*p* = 0.86	*p* = 0.53	*p* = 0.15	*p* = 0.10	*p* = 0.28
Calibration intercept	0.05	0.08	0.05	0.05	0.03	− 0.03	0.02
Calibration slope	0.84	0.79	0.86	0.80	0.82	0.89	0.88

This table shows the adjusted OR (CI) of each MSE metric as well as corresponding AUC, Nagelkerke R^2^, and Brier Scores. Furthermore, optimism-corrected performance estimates (AUC, Nagelkerke R^2^, and Brier Scores) estimated using bootstrapping are shown (internal validation). Lastly, the AUC, Hosmer–Lemeshow-goodness-of-fit and calibration slope/intercept are shown for assessment of external validity

^a^*ABP* mean arterial blood pressure, *AMP* intracranial pressure amplitude, *AUC* area under the curve, *CI* confidence interval, *CPP* cerebral perfusion pressure, *DBP* diastolic blood pressure, *HR* heart rate, *ICP* intracranial pressure, *MSE* multiscale entropy, *OR* odds ratio, *R*^2^ Nagelkerke R^2^, *SBP* systolic blood pressure

To assess internal validity, optimism-corrected performance estimates (AUC, Nagelkerke R^2^, and Brier Scores) were established using bootstrapping and are shown in Table 4 (middle panel). AUC optimism was at most 0.01 and R^2^ optimism was between 0.01 and 0.02. To assess external validity, the models were applied to the validation cohort describing discriminatory performance (AUC) and calibration using the Hosmer–Lemeshow-goodness-of-fit test and the calibration intercepts and slopes (Table 4, bottom panel). AUCs of the models when applied to the validation cohort were between 0.72 and 0.80. The Hosmer–Lemeshow-goodness-of-fit test found good model fits (test statistics were non-significant). The calibration slope was between 0.79 and 0.88 with the intercept being close to 0 in all cases.

### Ordinal multivariable analysis

To assess the effect of MSE on the outcome in form of an ordinal scale, a proportional odds logistic regression model adjusted for covariates was produced. The proportional odds assumptions were met for all MSE metrics and the common OR and *p*-values of the proportional odds regression are shown in Table 5. Common OR ranged between 0.79 and 0.88. Lastly, a sliding dichotomy approach was used to estimate the added value of MSE metrics when outcome was dichotomized based on individualized outcome prediction. The OR and p-values of the sliding dichotomy approach can be found in Table 5. Overall, OR ranged between 0.82 and 0.91.

**Table 5:** Ordinal analyses: proportional odds regression and sliding dichotomy.^a^

Proportional odds regression	MSE abp	MSE sbp	MSE dbp	MSE cpp	MSE hr	MSE icp	MSE amp
OR (CI)	0.81 (0.75–0.87)	0.79 (0.74–0.85)	0.82 (0.76–0.88)	0.85 (0.79–0.91)	0.83 (0.79–0.88)	0.86 (0.82–0.91)	0.88 (0.83–0.94)
*p*-value	2.9 e-9	3.6 e-11	2.5 e-7	1.5 e-5	3.0 e-10	1.7 e-7	4.1 e-5

Sliding dichotomy	MSE abp	MSE sbp	MSE dbp	MSE cpp	MSE hr	MSE icp	MSE amp
OR (CI)	0.82 (0.76–0.88)	0.82 (0.77–0.88)	0.82 (0.76–0.89)	0.89 (0.81–0.95)	0.87 (0.82–0.91)	0.89 (0.85–0.95)	0.91 (0.85–0.96)
*p*-value	1.9 e-7	3.4 e-8	1.2 e-6	0.001	2.6 e-7	1.7 e-4	0.003

^a^*ABP* mean arterial blood pressure, *AMP* intracranial pressure amplitude, *CI* confidence interval, *CPP* cerebral perfusion pressure, *DBP* diastolic blood pressure; *HR* heart rate, *ICP* intracranial pressure, *MSE* multiscale entropy, *OR* odds ratio, *SBP* systolic blood pressure

### Secondary analysis

Different further aspects of MSE were evaluated within the secondary analysis. Firstly, to evaluate the potential for early outcome prediction, MSE based on only the data acquired within the first 48 h after NCCU admission was evaluated. MSE sbp, MSE hr, and MSE icp all remained associated with outcome both when considered within univariable as well as multivariable and ordinal analyses (Additional file 1: C). After correction for the confounders (age, WFNS, mFisher, and occurrence of DCI) the OR of MSE sbp, MSE hr, and MSE icp were 0.87 (0.81–0.94), 0.86 (0.81–0.92), and 0.88 (0.83–0.95) per 1 step increase respectively. Overall, the models showed good discrimination with AUCs of 0.81 (0.75–0.85), 0.83 (0.77–0.87), 0.78 (0.70–0.85) for the multivariable logistic regression models including MSE sbp, MSE hr, and MSE icp respectively. The additional analyses can be found in Additional file 1: C.

In a second step, the MSE metrics MSE sbp, MSE hr, and MSE icp were evaluated against different clinical aspects and events (Additional file 1: D). Higher WFNS grade was associated with a decrease in all MSE metrics (MSE sbp: 25.4 ± 4.5 vs. 21.7 ± 4.2, *p* < 0.001; MSE hr: 24.6 ± 4.8 vs. 20.0 ± 5.1, *p* < 0.001; MSE icp: 21 ± 7 vs. 16 ± 6, *p* < 0.001 for low vs. high WFNS respectively). While no MSE metric was associated with mFisher, MSE icp was higher in patients who received coiling (19 ± 7) as compared to clipping (16 ± 6, *p* = 0.003). Conditions associated with or resulting from high ICP (cerebral edema, brain herniation) were associated with decreases in all three MSE metrics. Rebleeding and hydrocephalus on the other hand were only associated with a decrease in MSE sbp (rebleeding: 23.9 ± 4.6 vs. 20.0 ± 5.8, *p* = 0.008; hydrocephalus: 25.2 ± 5.1 vs. 22.4 ± 4.3, *p* < 0.001) and MSE hr (rebleeding: 23.1 ± 5.5 vs. 19.2 ± 6.7, *p* = 0.032; hydrocephalus: 24.1 ± 5.8 vs. 21.0 ± 5.2, *p* < 0.001) but not MSE icp. Similarly, DCI was associated with a decrease in MSE sbp (23.7 ± 5.0 vs. 22.5 ± 4.1, *p* = 0.039) and MSE hr (22.8 ± 5.6 vs. 20.2 ± 5.3, *p* < 0.001), but not MSE icp. The additional results can be found Additional file 1: D.

Lastly, the stability of MSE was assess by evaluating the change when including longer amounts of data as well as when comparing the results of the metrics to the duration of the measurement (Additional file 1: E). Starting from 3 to 6 h, stable MSE values could be found. There was no difference in absolute value of MSE compared to the duration of the recording when considering all patients.

## Discussion

Entropy, and in particular its multiscale version, MSE, builds on the previously described concept that physiological systems are regulated by multiple, interacting, mechanisms that collectively result in dynamically changing, irregular, fluctuations of biosignals across different temporal scales [14]. Lower MSE represents higher regularity of a system. While a “stable”, “regular” system would at first glance seemingly give the impression of being ‘healthy’, in reality, such a system is more rigid, with impaired capacity to counteract ever-present, random environmental triggers. The disease course of aSAH includes a variety of pathological processes necessitating continuous and rapid adjustments to equilibrate the system [27]. Patients that survive the initial hemorrhage remain at risk for developing secondary brain injury due to numerous pathophysiological cascades and complications. As part of the early brain injury, due to the initial hemorrhage, ICP rises either immediately (due to the volume of the bleeding itself [28]) or with a certain delay (i.e. resulting hydrocephalus [29] and/or brain edema). In the worst-case scenario, either one can lead to a relevant reduction in CPP. If the system fails to counteract this decrease in cerebral perfusion, this may lead to transient cerebral hypoxia or, ultimately, even infarction. Various injury cascades (upregulation of inflammatory pathways [30], coagulopathy [31]) further damage the system in case of cerebral hypoxia. A non-reactive system, represented consequently by low MSE, has been associated with higher pressure reactivity index (PRx) values supporting the notion that MSE represents the activity level of the physiological regulation systems, including cerebral blood flow autoregulation [9, 15]. While both metrics clearly share similar mechanisms, to equate MSE with cerebrovascular reactivity (as assessed using PRx) would be an oversimplification, considering Lu et al. also showed that PRx loses its predictive value when MSE is added to multivariable regression models for outcome prediction after traumatic brain injury [9].

The main driver of secondary brain injury in aSAH is DCI [3]. Although, DCI is the consequence of complex interacting pathophysiological sequelae, a well described and potentially reversible cause is vasospasm, which describes the narrowing of cerebral vessels [4]. Depending on the severity of such narrowing, ischemia or even infarction can occur. In aSAH, intact cerebral autoregulation is essential to counteract such dynamic reductions of vessel diameter by automatically and immediately increasing CPP. Autoregulation failure is detrimental, as symptomatic treatment (using vasoactive medications or intra-arterial spasmolysis) can only be initiated with a significant delay.

In addition to cerebral complications, aSAH leads to various systemic and most prominently cardiac complications [32, 33]. Both, myocardial ischemia and neurogenic stunned myocardium are found after aSAH leading to wall motion abnormalities and consequently reduced cardiac output. In the worst case, cardiac complications coincide with (or cause) pulmonary edema leading to further impairment of cardiac function and oxygenation [34–36]. Sufficient cardiac output is necessary to counteract impaired perfusion due to vasospasm. Cardiac output guided therapy is beneficial for managing cerebral oxygenation in patients with vasospasm [7, 37]. Myocardial injury might indeed be one cause of decreased entropy with previous reports describing good discrimination when assessing heart rate variability after aSAH and diagnosis of neurocardiogenic injury [17]. To date, the most commonly evaluated complexity related metric in aSAH is heart rate variability due to its simple determination based on short electrocardiograms [18]. Considering these results, it is not surprising that MSE hr was a predictor of outcome. However, variability and entropy cannot be used interchangeably due to the large number of metrics used for description some of which depend on distribution while others depend on specific patterns.

In aSAH, due to the complex nature and course of disease, NCCU monitoring plays a pivotal role for guiding treatment. Neurocritical care bioinformatics allow for the acquisition, integration, and synchronization of the various biosignals within the same environment, thereby permitting exploitation of advanced data-driven methods for guiding treatment and outcome prediction [38]. Yet, computational methods remain underutilized and are generally not readily available for direct bedside implementation. Commonly, single (at times arithmetic mean or worse, single snapshot) targets are used for guiding treatment, ignoring the potential benefits of complex integration. The results of this study underline the potential of advanced analytical tools for improving the understanding of the complex pathophysiology of multimodal monitoring dynamics after aSAH. However, relevant limitations exist.

## Limitations

Although this study included over 300 patients from 2 centres, they were all treated at highly specialized, high resource centers with relatively high patient volumes. On a similar note, patients underwent active treatment throughout their NCCU stay. It is unclear if, and to what extent interventions and complications are associated with changes in MSE. This is underlined by the results showing changes in MSE depending on various events. Either aspect, however, implies that the biosignals acquired do not represent the “natural” course of the disease, but patients undergoing active treatment. Due to the exploratory nature of this study, we did not explore time-trends of MSE, thus we cannot comment on the patterns of variability of MSE over the course of the NCCU stay (e.g. depending on intervention, medication or similar) or whether there were specific turning points (e.g. refractory ICP increase, DCI etc.). Overall, many complications and disease aspects were associated with changes in MSE and it is likely that the resulting metric represents a composite of various aspects. It is important to note that while the results were adjusted for the relevant and known outcome predictors age, clinical and imaging severity, and the important complication DCI, many other clinical variables are of importance when predicting outcome. To date, no multimodal monitoring based metric alone can or should replace clinical examination. However, metrics such as MSE should be seen as complementary allowing for additional physiology information.

## Conclusions

This study provides the first description of MSE as an outcome predictor after aSAH. MSE metrics and thereby complexity of physiological signals are independent, internally and externally valid predictors of 12 month outcome after aSAH. MSE decreases monotonically with worse outcomes and remains a valid outcome predictor when adjusting the outcome definition to the initial severity of disease and age. The promising results of this study warrant further investigation into the cause of the resulting complexity as well as its association with important and potentially preventable complications (i.e. vasospasm and DCI). Of particular importance will be the assessment of time-trends and the evaluation of intraindividual episodes of decreased entropy and their association to specific events. Promising targets for such analysis are MSE sbp and MSE hr since neither requires continuous neuromonitoring and can therefore be applied to the whole aSAH population.

### Supplementary Information

**Additional file 1.** Supplementary data describing data coverage (A), physiological metrics (B), and the results of the secondary analyses (C-E).

## Data Availability

The processed data is available upon reasonable request by the corresponding author.

## Abbreviations

aSAH: Aneurysmal subarachnoid hemorrhage
ABP: Mean arterial blood pressure
AMP: Intracranial pressure amplitude
AUC: Area under the curve
CI: Confidence interval
CPP: Cerebral perfusion pressure
DBP: Diastolic blood pressure
DCI: Delayed cerebral ischemia
GOSE: Glasgow outcome scale extended
HR: Heart rate
ICP: Intracranial pressure
mFisher: Modified Fisher scale
MSE: Multiscale entropy
NCCU: Neurocritical care unit
NPV: Negative predictive value
OR: Odds ratio
PPV: Positive predictive value
ROC: Receiver operating characteristic
SBP: Systolic blood pressure
WFNS: World Federation of Neurosurgical Societies grading scale
