# Supplementary material for: Predicting outcome after aneurysmal subarachnoid hemorrhage by exploitation of signal complexity: a prospective two-center cohort study
Source: Crit Care. 2024 May 14;28:163. doi: 10.1186/s13054-024-04939-7 (PMC11092006; doi:10.1186/s13054-024-04939-7)

# **SUPPLEMENT**

**Supplement A. Data coverage.** The data coverage is shown in form of boxplots (length), histograms (frequency per length), and density (frequency of data depending on day post hemorrhage with day 0 being the day of initial hemorrhage) for the derivation cohort (A-C) and the validation cohort (D-F). On average, close to 10 days of high-resolution monitoring data was available for ABP, HR, and ICP (A, D). The distribution of available data durations per patient was similar in the derivation (B) and validation (E) cohorts with a sharp decrease at around 340 hours (i.e. 14 days). Within the density plots (C, F) a distinct peak in availability of data between day 3 and 14 after the initial hemorrhage (day 0) can be seen in both the derivation (C) and validation (E) cohorts. The median time between the initial hemorrhage and admission to the NCCU/start of multimodality monitoring was 18 hours in the derivation and 31 hours in the validation cohort.


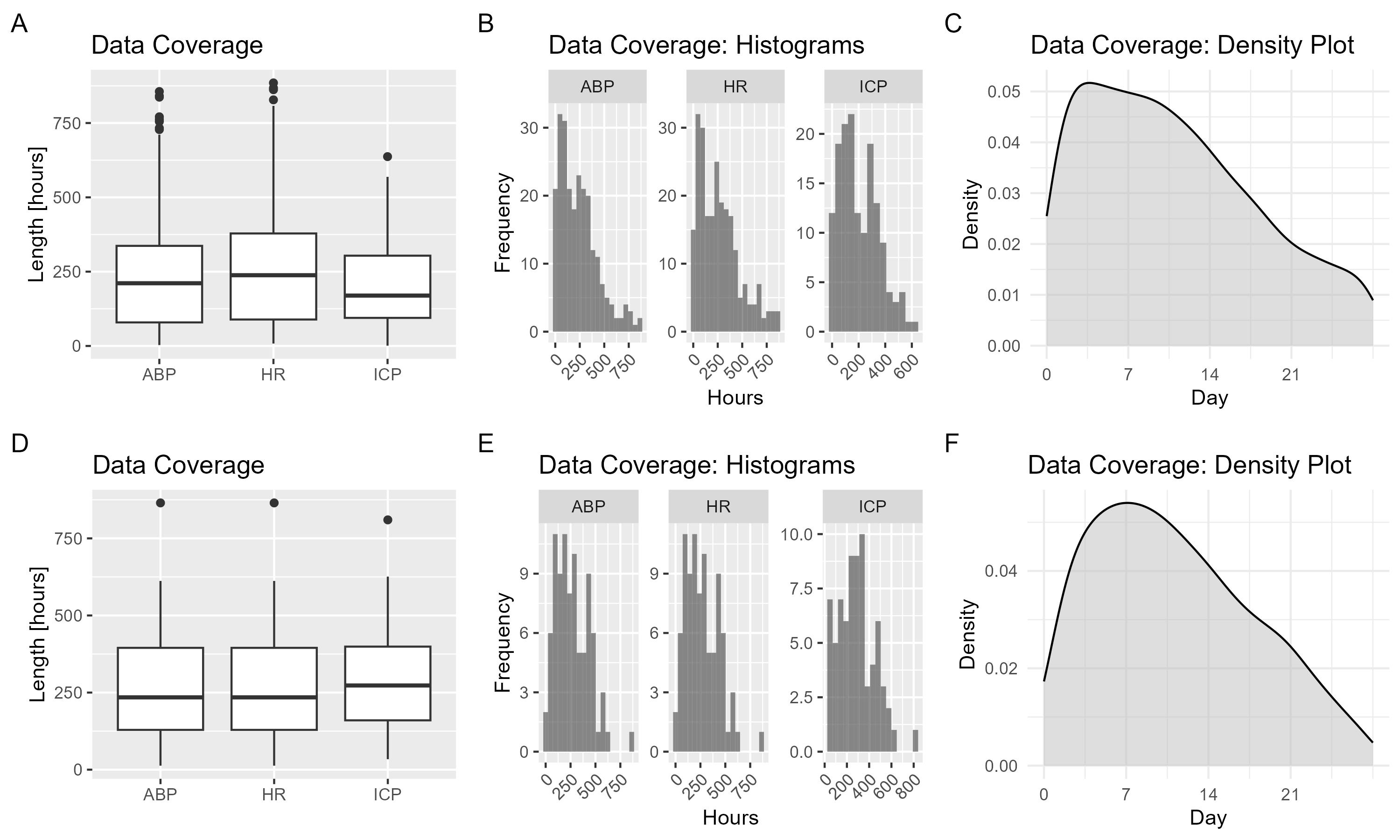


**
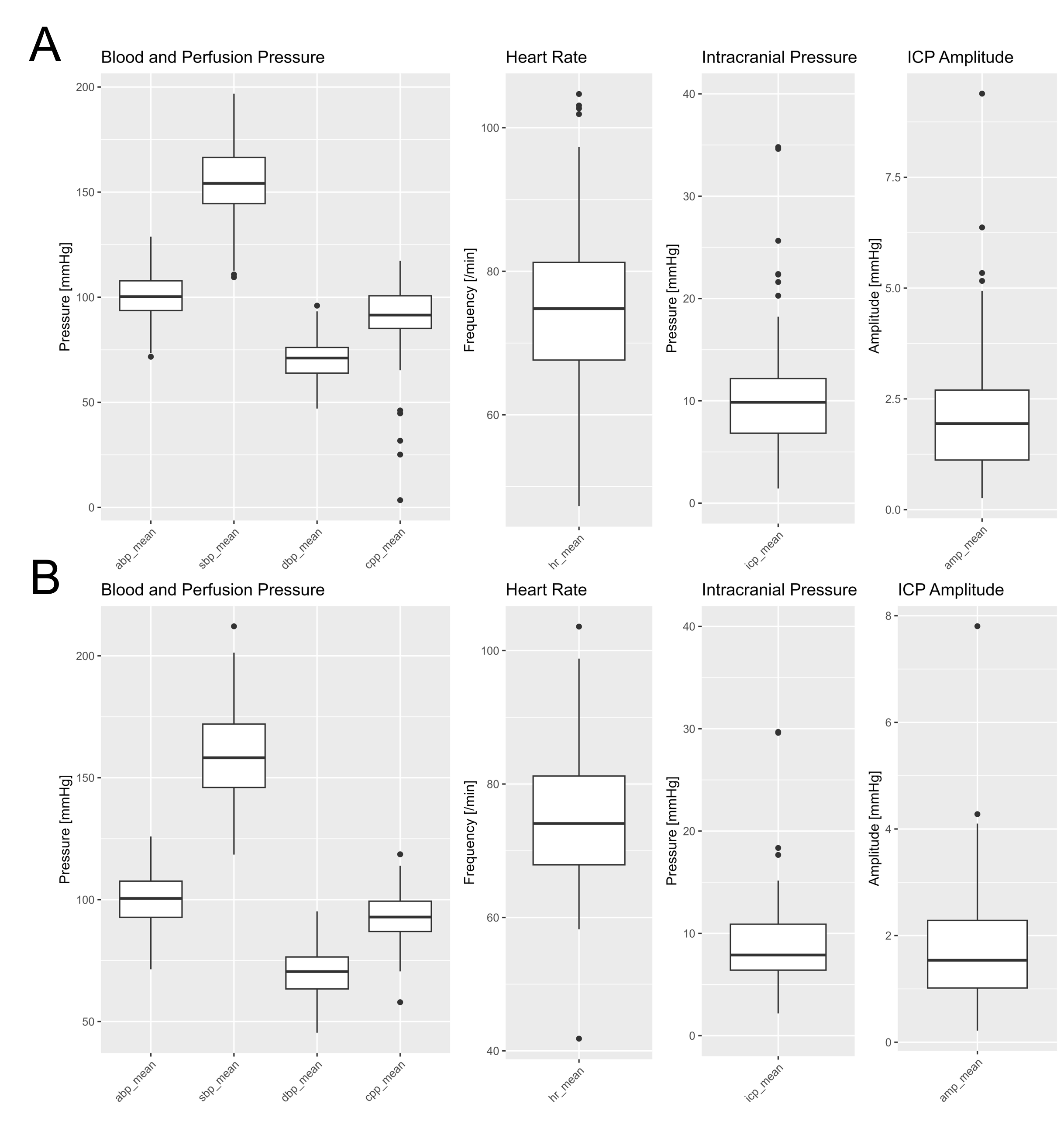
Supplement B. Physiological metrics.** Average mean/systolic/diastolic blood pressure, cerebral perfusion pressure, intracranial pressure and intracranial pressure amplitude are shown for both the derivation (A) and validation (B) cohorts.


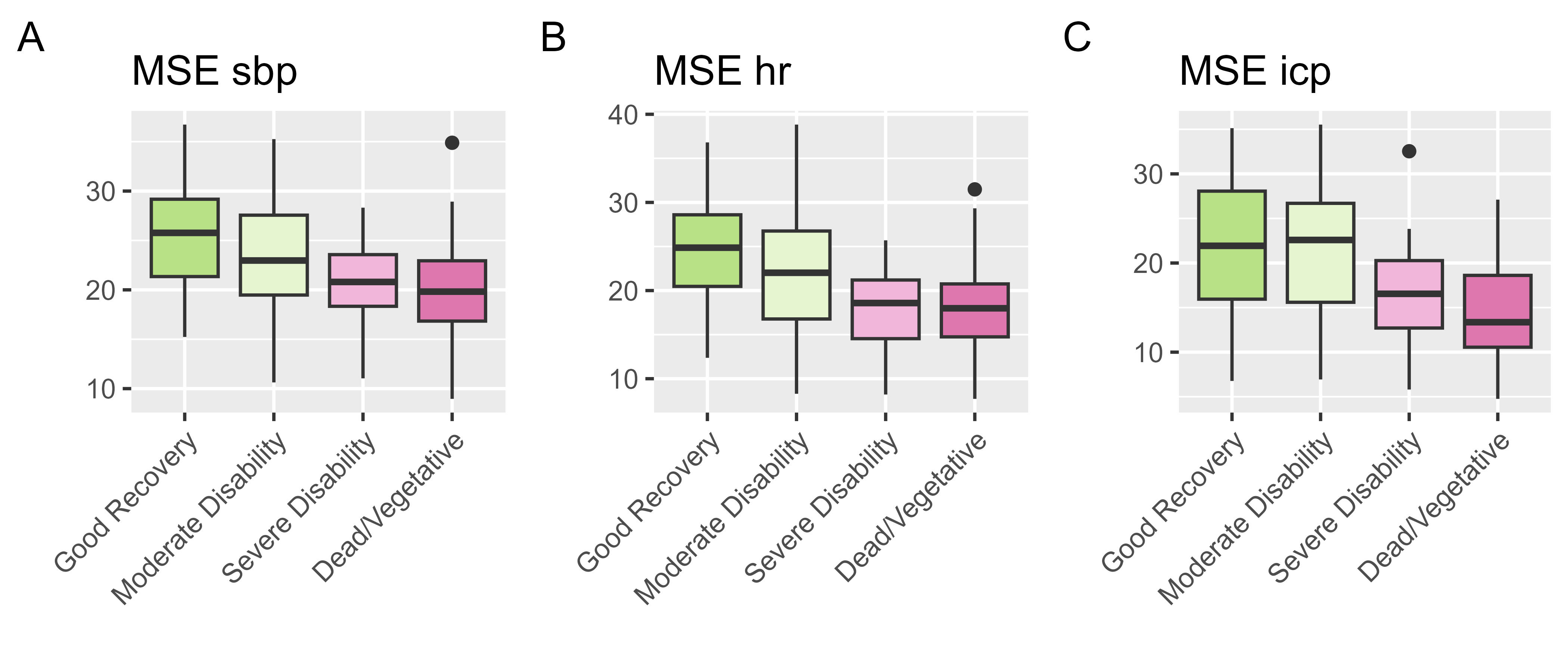
**Supplement C. MSE within the first 48 hours.** In order to evaluate whether early outcome prediction is possible, MSE was analyzed as part of the secondary analysis including only the data acquired within the first 48 hours and evaluating only the most promising metrics MSE sbp, MSE hr, and MSE icp. When evaluated within the univariable analysis, MSE sbp (A), MSE hr (B), and MSE icp (C) all differentiated between the different outcome groups (Figure, Table 1).

| **Table 1. P values of ANOVA comparing ordinal outcome and MSE metrics** | | | |
| --- | --- | --- | --- |
|  | **MSE sbp** | **MSE hr** | **MSE icp** |
| **Good vs. Moderate** | 0.049 | 0.022 | 0.233 |
| **Good vs. Severe** | <0.001 | <0.001 | 0.015 |
| **Good vs. Dead/Vegetative** | <0.001 | <0.001 | <0.001 |
| **Moderate vs. Severe** | 0.14 | 0.0049 | 0.021 |
| **Moderate vs. Dead/Vegetative** | 0.0051 | <0.001 | <0.001 |
| **Severe vs. Dead/Vegetative** | 0.081 | 0.192 | 0.634 |

To evaluate the independence of the metrics, they were then corrected for the predefined covariates (age, WFNS, mFisher, and occurrence of DCI) within multivariable logistic regression models or ordinal models (proportional odds regression, sliding dichotomy). MSE sbp, MSE hr, and MSE icp remained significant, independent predictors of outcome (Table 2). The achieved AUCs for the multivariable regression models were 0.81, 0.83, and 0.78 for MSE sbp, MSE hr, and MSE icp respectively.

| Table 2. Multivariable and ordinal covariate adjusted models | | | |
| --- | --- | --- | --- |
| **Multivariable Logistic Regression** | **MSE sbp** | **MSE hr** | **MSE icp** |
| OR (CI) | 0.87 (0.81-0.94) | 0.86 (0.81-0.92) | 0.88 (0.83-0.95) |
| AUC (CI) | 0.81 (0.75-0.85) | 0.83 (0.77-0.87) | 0.78 (0.70-0.85) |
| p-value | <0.001 | <0.001 | <0.001 |
|  |  |  |  |
| **Proportional Odds Regression** | **MSE sbp** | **MSE hr** | **MSE icp** |
| OR (CI) | 0.90 (0.85-0.95) | 0.90 (0.86-0.94) | 0.91 (0.87-0.96) |
| p-value | <0.001 | <0.001 | <0.001 |
|  |  |  |  |
| **Sliding Dichotomy** | **MSE sbp** | **MSE hr** | **MSE icp** |
| OR (CI) | 0.90 (0.86-0.95) | 0.91 (0.86-0.95) | 0.92 (0.87-0.97) |
| p-value | <0.001 | <0.001 | 0.002 |

**Supplement D. MSE depending on clinical aspects.** To elucidate whether the found values of MSE were associated with relevant clinical events, additional univariable statistical analyses (t-test) were performed evaluating MSE sbp, MSE hr, and MSE icp depending on clinical events and aspects (Table 1). Furthermore, values of MSE were plotted against the raw metric (ABP, HR, ICP) to evaluate possible relationships (Figure A-C).

| Table 1. MSE vs. clinical events | | | | | | | | | |
| --- | --- | --- | --- | --- | --- | --- | --- | --- | --- |
|  | **MSE sbp** (mean±SD) | | | **MSE hr** (mean±SD) | | | **MSE icp** (mean±SD) | | |
|  | **1-2** | **3-5** | **p** | **1-2** | **3-5** | **p** | **1-2** | **3-5** | **p** |
| ***WFNS*** | 25.4±4.5 | 21.7±4.2 | <0.001 | 24.6±4.8 | 20.0±5.1 | <0.001 | 21±7 | 16±6 | <0.001 |
|  | **1-2** | **3-4** | **p** | **1-2** | **3-4** | **p** | **1-2** | **3-4** | **p** |
| ***mFisher*** | 24.7±5.2 | 23.2±4.6 | 0.089 | 22.9±5.6 | 22.0±5.5 | 0.3 | 18±8 | 17±6 | 0.7 |
|  | **Clipping** | **Coiling** | **p** | **Clipping** | **Coiling** | **p** | **Clipping** | **Coiling** | **p** |
| ***Treatment*** | 23.5±4.5 | 23.4±4.9 | 0.9 | 22.1±5.6 | 22.3±5.5 | 0.9 | 16±6 | 19±7 | 0.003 |
|  |  |  |  |  |  |  |  |  |  |
|  | **Absent** | **Present** | **p** | **Absent** | **Present** | **p** | **Absent** | **Present** | **p** |
| ***Rebleeding*** | 23.9±4.6 | 20.0±5.8 | 0.008 | 23.1±5.5 | 19.2±6.7 | 0.032 | 18.7±6.4 | 16.8±7.0 | 0.4 |
| ***Intracranial hypertension†*** | 22.9±3.9 | 16.6±4.6 | <0.001 | 22.3±4.9 | 14.6±3.6 | <0.001 | 19.0±6.4 | 13.0±4.6 | 0.005 |
| ***Cerebral edema**** | 24.1±4.7 | 21.7±4.4 | 0.004 | 23.4±5.4 | 20.2±5.8 | 0.003 | 19.3±6.5 | 14.7±5.2 | <0.001 |
| ***Herniation**** | 24.2±4.5 | 20.7±5.0 | <0.001 | 23.6±5.2 | 18.8±6.1 | <0.001 | 19.4±6.2 | 14.7±6.2 | <0.001 |
| ***Hydrocephalus*** | 25.2±5.1 | 22.4±4.3 | <0.001 | 24.1±5.8 | 21.0±5.2 | <0.001 | 18±7 | 17±7 | 0.5 |
| ***DCI*** | 23.7±5.0 | 22.5±4.1 | 0.039 | 22.8±5.6 | 20.2±5.3 | <0.001 | 18±7 | 16±6 | 0.3 |
| ***Vasospasm*** | 23.8±5.4 | 23.1±4.3 | 0.14 | 22.7±5.9 | 21.7±5.4 | 0.052 | 17±7 | 18±6 | 0.12 |
| ***Seizures**** | 24.4±4.9 | 22.2±4.1 | <0.001 | 23.9±5.4 | 20.7±5.4 | <0.001 | 18.9±6.5 | 17.9±6.4 | 0.4 |


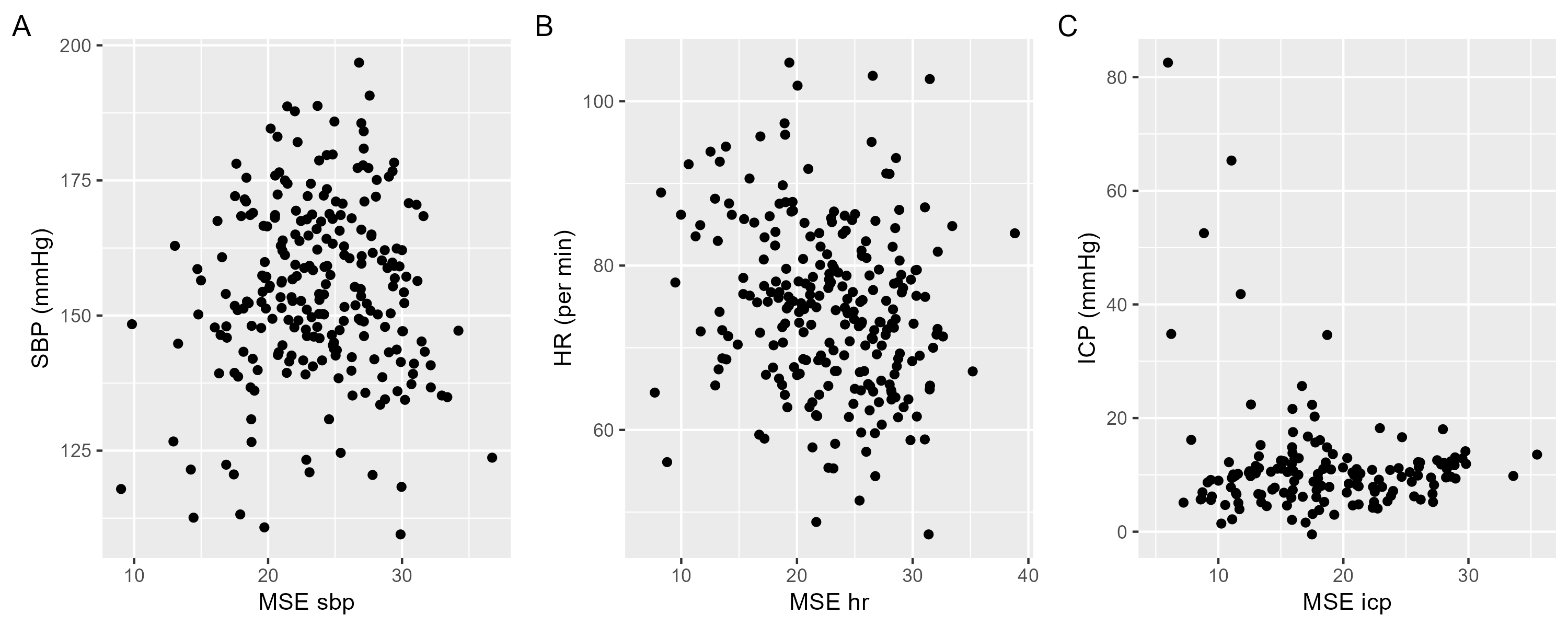


*only available in derivation cohort, † ICP >20 mmHg

**Supplement E. Effect of Duration of MSE.** MSE is based on sample entropy which was first described and evaluated by Richman et al.^1^. We explored (in a single patient) the effect of increasing number of samples (corresponding to 1-24 hours of data) on the absolute value of MSE abp (A) and the correlation (Pearson correlation coefficient) between corresponding sections of data (B). Consistent with the explorations by Richman, we found stable results with a duration of around 1000 samples or more (i.e. 3-6h). Furthermore, there was no association between the duration of recording and the level of the derived MSE (C-E).

1. Richman JS, Moorman JR. Physiological time-series analysis using approximate entropy and sample entropy. American journal of physiology-heart and circulatory physiology 2000.


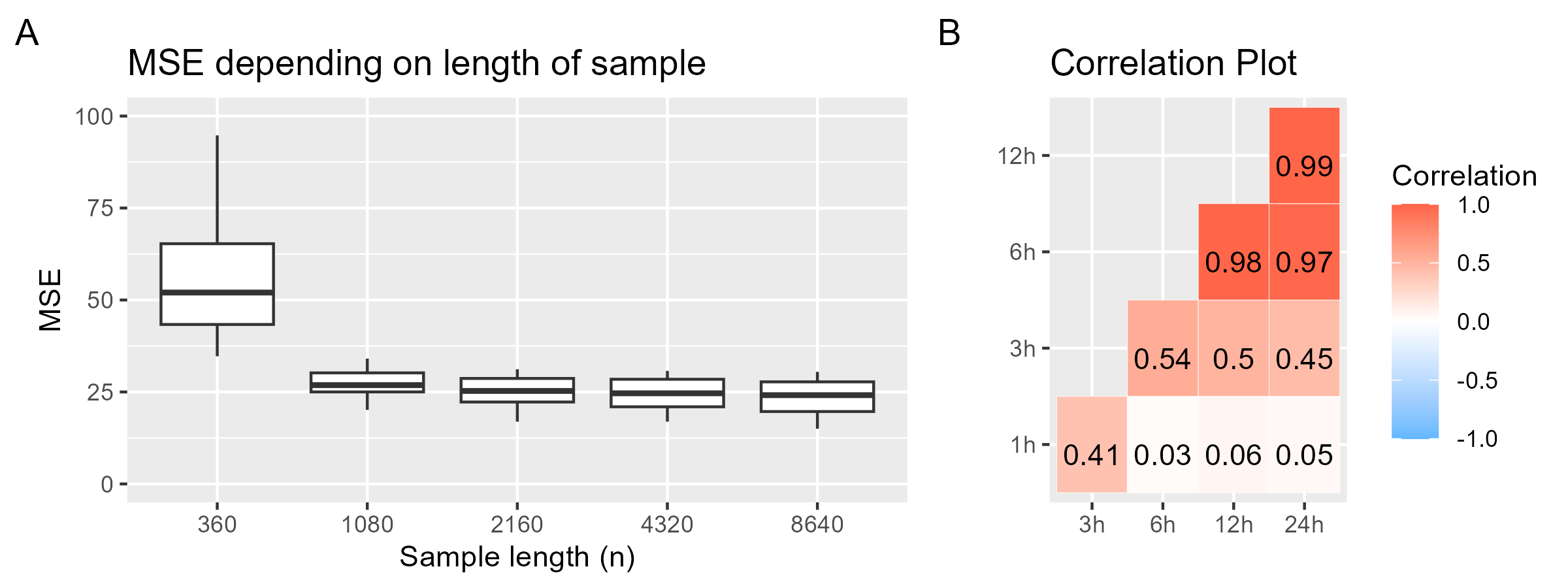

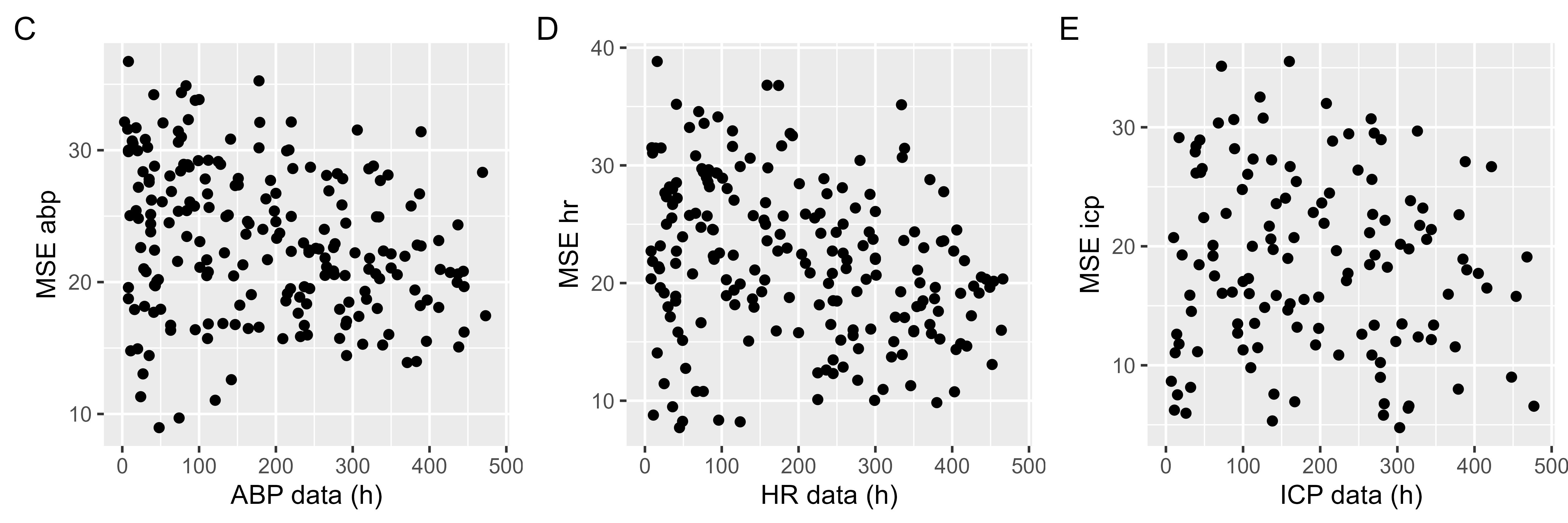

Supplement: Supplementary file 1 — Additional file 1. Supplementary data describing data coverage (A), physiological metrics (B), and the results of the secondary analyses (C-E). [file 13054_2024_4939_MOESM1_ESM.docx]
